# Involvement of *tumor suppressor candidate 3* in mouse survival, craniofacial, and cortical development

**DOI:** 10.1093/g3journal/jkag224

**Published:** 2026-08-21

**Authors:** Paul P R Iyyanar, Andrew Vontell, Ammar Husami, Ethan Sperry, K Nicole Weaver, Rolf W Stottmann

**Affiliations:** Steve and Cindy Rasmussen Institute for Genomic Medicine, Abigail Wexner Research Institute, Nationwide Children's Hospital, Columbus, OH 43205, United States; Steve and Cindy Rasmussen Institute for Genomic Medicine, Abigail Wexner Research Institute, Nationwide Children's Hospital, Columbus, OH 43205, United States; Division of Human Genetics, Cincinnati Children's Hospital Medical Center, Cincinnati, OH 45229, United States; Division of Human Genetics, Cincinnati Children's Hospital Medical Center, Cincinnati, OH 45229, United States; Division of Human Genetics, Cincinnati Children's Hospital Medical Center, Cincinnati, OH 45229, United States; Steve and Cindy Rasmussen Institute for Genomic Medicine, Abigail Wexner Research Institute, Nationwide Children's Hospital, Columbus, OH 43205, United States; Department of Pediatrics, The Ohio State University College of Medicine, Columbus, OH 43210, United States

**Keywords:** Tusc3, craniofacial, mandible, cleft palate, glycosylation, cortex, RNA-seq

## Abstract

Tumor suppressor candidate 3 is an integral component of the oligosaccharyltransferase complex and is required for the N-glycosylation of proteins. While tumor suppressor candidate 3 has been implicated in cancer and autosomal recessive non-syndromic intellectual disability, some patients also exhibit distinct facial features. However, the role of *Tusc3* in craniofacial development is unknown. Here, we report a patient presenting with cleft lip and palate who was found to carry a homozygous variant in the *TUSC3* promoter region in addition to a maternally inherited chromosomal duplication. In order to evaluate a potential role for *TUSC3* in craniofacial development, we analyzed *Tusc3* deletion mouse mutants from the International Mouse Phenotyping Consortium (IMPC). Initial IMPC phenotyping data suggested that a subset of *Tusc3* deletion mice could develop cleft palate, micrognathia, and aglossia. We further identified that *Tusc3* is expressed in the craniofacial and brain regions during embryonic development. Upon characterizing the *Tusc3* deletion line, we observed that most homozygous mutants exhibit preweaning lethality. While craniofacial defects occur at a very low frequency in the deletion mutants, we identified a modest reduction in cortical area. Furthermore, RNA-seq analysis surprisingly revealed that no other genes in the developing brain or face were significantly affected upon loss of *Tusc3*. These findings suggest that *Tusc3* can contribute to congenital malformations in both craniofacial and cortical development.

## Introduction

*Tumor suppressor candidate 3* (*Tusc3*) was originally identified for its role in metastatic cancer by physical mapping to the short arm of chromosome 8 (Bova et al. 1996; MacGrogan et al. 1996). The TUSC3 protein has several functions, including facilitating cellular uptake of magnesium and protein glycosylation (Zhou and Clapham 2009; Mohorko et al. 2014). TUSC3 is localized to the endoplasmic reticulum and acts as an integral component of the oligosaccharyl transferase complex, which is responsible for N-linked protein glycosylation (Mohorko et al. 2014). TUSC3 is hypothesized to modulate N-linked glycosylation efficiency for a subset of glycoproteins, thereby influencing protein folding and ER homeostasis (Mohorko et al. 2014). *Tusc3* expression is enriched in adipose tissue, colon, cervix, lung, ovary, placenta, and prostate in humans (Zhou and Clapham 2009). Morpholino-mediated knockdown of *Tusc3* in zebrafish resulted in defects in maturation and hatching (Zhou and Clapham 2009).

*TUSC3* is essential for neurodevelopment and homozygous pathogenic variants in *TUSC3* have been implicated in non-syndromic autosomal recessive intellectual disability (intellectual developmental disorder, autosomal recessive 7; MIM: 611093) (Garshasbi et al. 2008; Molinari et al. 2008; Garshasbi et al. 2011; Mhamdi et al. 2011; Loddo et al. 2013; Ghadami et al. 2015; Zhang et al. 2015; Al-Amri et al. 2016). These patients exhibited a deficit in memory, learning, and cognitive function likely due to defective N-glycosylation leading to ER stress (Mohorko et al. 2014). In some cases, *TUSC3* mutations are also associated with seizures, microcephaly, and in rare cases with short stature (Garshasbi et al. 2008; Al-Amri et al. 2016). TUSC3 patients were also reported to have dysmorphic facial features characterized by a long face, deep-set eyes, a thin philtrum, down-slanting palpebral fissures, apparent hypertelorism, and pointed chin, with one case of tied tongue (ankyloglossia) (Garshasbi et al. 2011; Al-Amri et al. 2016).

Loss of *TUSC3* through genetic deletion or promoter hypermethylation is linked to increased cell proliferation, migration invasion, epithelial-to-mesenchymal transition, and altered AKT and Hedgehog pathway signaling in multiple epithelial cancers (Vanhara et al. 2013; Horak et al. 2014; Mohorko et al. 2014; Kratochvilova et al. 2015; Fan et al. 2016; Jiang et al. 2016; Gu et al. 2017; Taniue et al. 2020). Here, we report a patient with a rare variant in the *TUSC3* promoter region with unilateral cleft lip. We used the *Tusc3* deletion mouse model to study the role of this gene in craniofacial development.

## Materials and methods

### Human subjects and variant analysis

Genetic analysis was performed under a protocol approved by the Cincinnati Children's Hospital Medical Center IRB (CCHMC IRB_2014_3789). Written informed consent/assent was obtained from all participants. Whole-genome sequencing was done at BGI America USA to an average depth of at least 30× with standard protocols. VCF-format small SNPs and InDels were annotated and filtered using standardized protocols. The variant annotation and interpretation analyses were generated through the use of Ingenuity Variant Analysis software from QIAGEN, Inc. Variants were filtered based on population frequencies. Subsequently, variants known or predicted to have semantic similarity with micrognathia and cleft palate were discovered using Phenotype Driven Ranking (PDR) filter.

### Animals

*Tusc3 deletion* (*Tusc3^del^*) mice were obtained from MMRRC (C57BL/6N-*Tusc3^em1(IMPC)J^*/Mmucd; Stock number: 050255-UCD). The *Tusc3^del^* resulted in the deletion of 106 bp spanning the second exon, leading to an amino acid change after residue 81 and early truncation 2 amino acids later. The breeding was carried out on the C57BL/6J background. Mice were housed with a 12 h light/12 h dark cycle with food and water ad libitum. For timed mating, noon of the day of the vaginal plug was considered embryonic day (E) 0.5. Mouse euthanasia was performed in a carbon dioxide chamber followed by secondary cervical dislocation. All animal work procedures were performed following recommendations in the Guide for Care and Use of Laboratory Animals by the National Institutes of Health and approved by the Institutional Animal Care and Use Committee (IACUC) at Nationwide Children's Hospital (AR21-00067).

### International mouse phenotyping consortium

Initial phenotypic characterization was carried out by analyzing the serial sections of the micro-computed tomography (micro-CT) pictures from the *Tusc3* mutant mice on the International Mouse Phenotyping Consortium (IMPC; Birling et al. 2021; Wilson et al. 2026) website (https://www.mousephenotype.org/). MicroCT images were obtained from the International Mouse Phenotyping Consortium (IMPC; mousephenotype.org) and are reproduced under the Creative Commons Attribution 4.0 International (CC BY 4.0) license.

### RNAscope

RNA in situ hybridization with RNAscope probes was carried out using standard procedures as previously described (Blizzard et al. 2021). Embryos were dissected at ages E14.5 and E18.5 and were fixed in formalin for 16 to 24 h. The tissue was washed in PBS, then dehydrated and embedded in paraffin and was sectioned on the microtome at 5 µm, placed on SuperFrost slides, then baked at 60 °C for 1 h. Hybridization and amplification steps were performed using the HybEZ oven (Advanced Cell Diagnostics [ACD], Newark, CA, United States) set at 40 °C. A manual assay protocol was followed using RNAscope Multiplex Fluorescent Reagent Kit V2 (323100), TSA Cyanine 3 Fluorophores (NEL744001KT) at 1:750, and a C1 *Tusc3* probe made to order (Catalog # 1274161-C1). The ACD RNAscope negative control probe dapB (Advanced Cell Diagnostics, Newark, CA, United States, Cat# 310043), targeting the bacterial Bacillus subtilis dihydrodipicolinate reductase transcript, was included as a negative control to evaluate nonspecific background staining in mammalian tissue sections. All reagents were purchased from ACD.

### Skeletal preparations

Skeletal preparations were carried out using Alcian Blue and Alizarin Red to stain cartilage and bone, respectively, as per standard procedures. Briefly, animals were skinned, eviscerated, and fixed for 2 d in 95% ethanol. Samples were stained overnight at room temperature in 0.03% (w/v) Alcian Blue solution (Sigma-Aldrich, A3157) containing 80% ethanol and 20% glacial acetic acid and were destained in 95% ethanol for 24 h followed by preclearing in 1% KOH overnight at room temperature. Skeletons were then stained for calcium in bone matrix in 0.005% Alizarin Red solution overnight (Sigma-Aldrich, A5533) containing 1% KOH. Samples were then cleared in 20% glycerol/1% KOH solution for 24 h. Finally, they were transferred to 50% glycerol/50% ethanol for photography. Skeletal preparations were imaged using a Zeiss Discovery.V12 Stereoscope.

### Cortex measurement

Cortical surface area was measured from whole-mount brains at E18.5 and postnatal day 1 (P1) from images acquired with a Zeiss Discovery.V12 Stereoscope.

### RNA-seq

E18.5 brains and craniofacial tissues were dissected, snap-frozen, and stored at −80 °C until use. A total of 3 *Tusc3^wt/wt^* and 3 *Tusc3^del/del^* mutant heads were dissected. RNA samples were isolated and subjected to paired-end bulk RNA sequencing at the Genomic Services Laboratory in the Steve and Cindy Rasmussen Institute for Genomic Medicine at Nationwide Children's Hospital (RRID:SCR_017840). Differential gene expression analysis was performed using the DESeq2 package (version 1.26.0) in R (version 4.3.1). Raw gene-level count matrices were used as input and normalized using DESeq2's median-of-ratios method to account for differences in sequencing depth and RNA composition across samples. Differential expression was assessed using a negative binomial generalized linear model, with dispersion parameters estimated for each gene and empirical Bayes shrinkage applied to improve variance estimates. Wald tests were used to evaluate statistical significance of differential expression between experimental groups. Resulting *P*-values were adjusted for multiple hypothesis testing using the Benjamini–Hochberg false discovery rate (FDR) procedure. Genes with an adjusted *P*-value (FDR) < 0.10 and an absolute log2 fold change > 1.5 were considered significantly differentially expressed. Rank-based pathway enrichment analysis was performed using preranked lists of all expressed genes from brain and craniofacial tissues based on mutant vs control log2 fold-change values. Enrichment was assessed against KEGG, Reactome, WikiPathways, and Gene Ontology Biological Process databases. Pathways were evaluated using adjusted *P*-values following correction for multiple testing, with adjusted *P* < 0.25 considered significant.

### Statistical analysis

All statistical analyses were carried out using Prism 10 software (GraphPad Software, Boston, MA). One-way ANOVA followed by Tukey's multiple comparison test was used to determine statistical significance between all groups.

All materials can be found in the Reagents Table (Supplementary Table 4).

## Results

### A *TUSC3* variant in a patient with craniofacial anomalies

As part of a larger effort to identify novel genetic variants in patients with congenital craniofacial anomalies, we sequenced the unaffected parents and a female who was enrolled for her clinical presentation of left unilateral cleft lip, alveolar cleft, deep-set eyes, wide palpebral fissures, and smooth philtrum. A neurologic exam demonstrated hypotonia, and subsequent brain imaging showed a thin corpus callosum with right-sided heterotopia and mildly diminished white matter. The patient was noted to have a mild developmental delay most affecting speech and language (including a diagnosed speech apraxia). Previous clinical testing included a SNP microarray (CCHMC) which identified a duplication of uncertain significance located at 1p34.3-p34.2 (3.0 Mb), which was subsequently identified to likely be of maternal origin per clinical fluorescent in situ hybridization. Thus, the individual reported here exhibited some of the previously reported *TUSC3* phenotypes such as developmental delay and facial dysmorphism, including deep-set eyes, thin philtrum, and abnormal palpebral fissures. However, cleft lip/palate have not been previously reported (Table 1).

**Table 1.** *TUSC3* patients’ clinical presentations.

HPO term	HPO ID	Patient reported here	Garshasbi et al. 2008	Garshasbi et al. 2011	Khan et al. 2011	Al-Amri et al. 2016	Gumus 2020
Intellectual disability	HP:0001249	−	+	+	+	+	+
Global developmental delay	HP:0001263	+	−	−	+	2/5	+
Speech delay	HP:0000750	−	−	+	+	2/5	+
Microcephaly	HP:0000252	−	−	+	−	3/4	−
Facial dysmorphism (abnormal facial shape)	HP:0001999	+	−	−	−	4/4	+
Short stature	HP:0004322	−	−	−	1/6	3/4	−
Ankyloglossia	HP:0010296	−	−	+	−	1/4	−
Cleft lip; cleft palate	HP:0000204; HP:0000175	+	−	−	−	−	−

+, reported; −, absent.

Genome sequencing identified a homozygous duplication upstream of the *tumor suppressor candidate 3* (*TUSC3*) gene, which is predicted to cause promoter loss: Chr8(GRCh37):g.15396876_15396879dup; NM_006765.4(TUSC3):c.-1064_-1061dup (Fig. 1, a and b). Both parents are heterozygous for this variant. Genome sequencing also confirmed maternal inheritance of the chromosome 1 duplication.

**Fig. 1.**
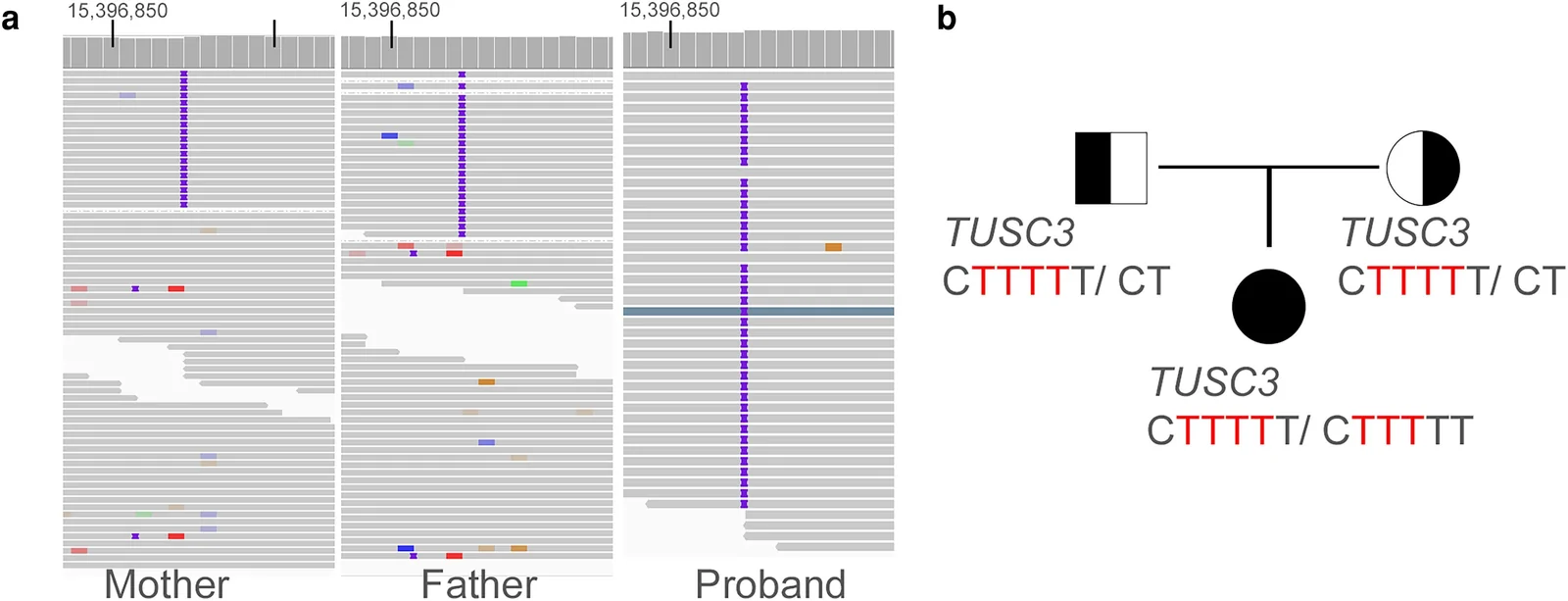
TUSC3 patients. a) IGV viewer shows the insertion of bases in the TUSC3 promoter region. The parents are heterozygous for this insertion, and the proband is homozygous. b) Family pedigree.

### *Tusc3* mutant phenotypes based on data from the International Mouse Phenotyping Consortium

The International Mouse Phenotyping Consortium (IMPC) has created a null allele of the *Tusc3* ortholog in mice (*Tusc3^em1(IMPC)J^*, referred to from here on as *Tusc3^del^*) and reported a preweaning lethality phenotype in the mice, with only approximately 3% of homozygous surviving (*n* = 1/33). At embryonic day (E) 18.5, homozygous *Tusc3^del/del^* mutants exhibited an abnormal body size (2/15) and abnormal body wall phenotype (2/8). Additionally, the IMPC also noted *Tusc3^del/wt^* heterozygous mutants with persistence of the hyaloid vasculature at the early adult stage (*n* = 10; *P* < 0.0001).

We analyzed images of *Tusc3* mutants provided by the IMPC website and identified craniofacial abnormalities in a subset of mutants. At E18.5, *Tusc3^del/del^* mutants exhibited a hypoplastic mandible (Fig. 2, a and b). Micro-CT images of the *Tusc3^del/del^* mutants further revealed the presence of cleft palate and aglossia of the tongue (Fig. 2, c to f; *n* = 1/8). Interestingly, the micro-CT images revealed that instead of bilateral palatal shelves fusing with each other, the palatal shelves of the *Tusc3^del/del^* mutants ectopically fused to the nasal structures (Fig. 2, e and f). These findings together with the clinical presentation of the patient suggest that *Tusc3* may contribute to craniofacial development.

**Fig. 2.**
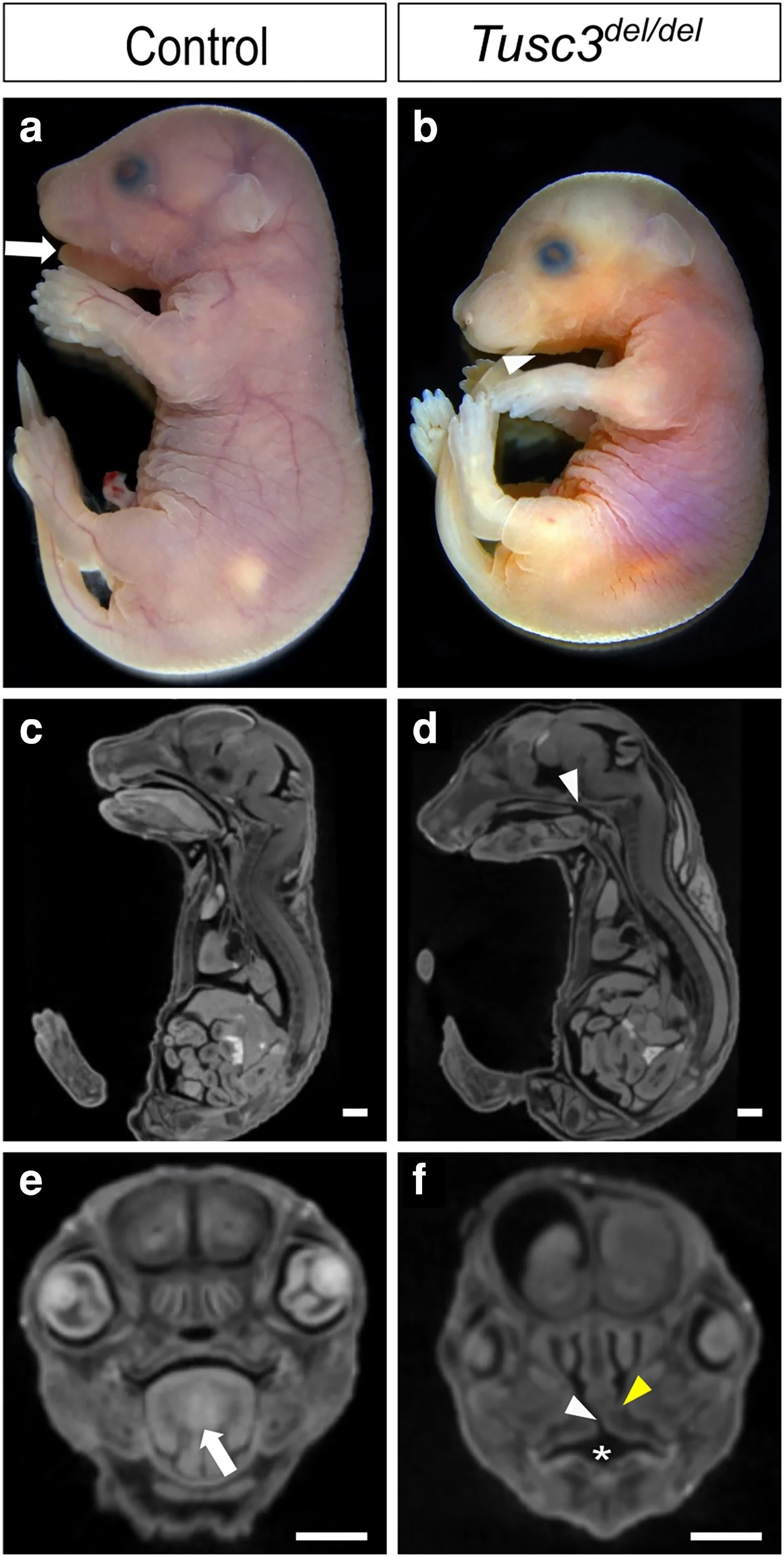
*Tusc3^del/del^* phenotype from IMPC data. a, b) Lateral views of whole-mount wildtype (a) and *Tusc3^del/del^* (b) embryos at E18.5. Arrow in a points to the normal mandible in the wildtype, whereas arrowhead in b points to the short mandible in the mutants. c to f) Sagittal (c, d) and frontal (e, f) views from micro-CT images of wildtype (c, e), and *Tusc3^del/del^* (d, f) embryos at E18.5. White arrowheads in d and f point to the cleft palate in *Tusc3^del/del^* embryos. Arrow in e points to the normal tongue in wildtype, while asterisk in f points to the absence of a tongue in *Tusc3^del/del^* embryos. Yellow arrowhead in f points to the palatal shelf fusing to nasal region. Scale bars, 1 mm.

### *Tusc3* expression pattern during development

To further characterize the role of *Tusc3*, we carried out RNA in situ hybridization with RNAscope probes to determine the expression of *Tusc3* during embryonic development at E18.5 and E14.5. We observed strong expression of *Tusc3* in the brain region, retina, olfactory epithelia, and trigeminal ganglia at E18.5 (Fig. 3, a and b). Additionally, strong expression was noted in the developing upper and lower molar tooth buds, the middle ear, and the epithelia of various craniofacial structures (Fig. 3, a and b). At E14.5, *Tusc3* exhibited strong expression in the developing cortex, trigeminal ganglia, and middle ear region (Fig. 3c). These data demonstrate that *Tusc3* is expressed in developing brain and craniofacial tissues during embryogenesis, supporting a potential role in cortical and craniofacial development.

**Fig. 3.**
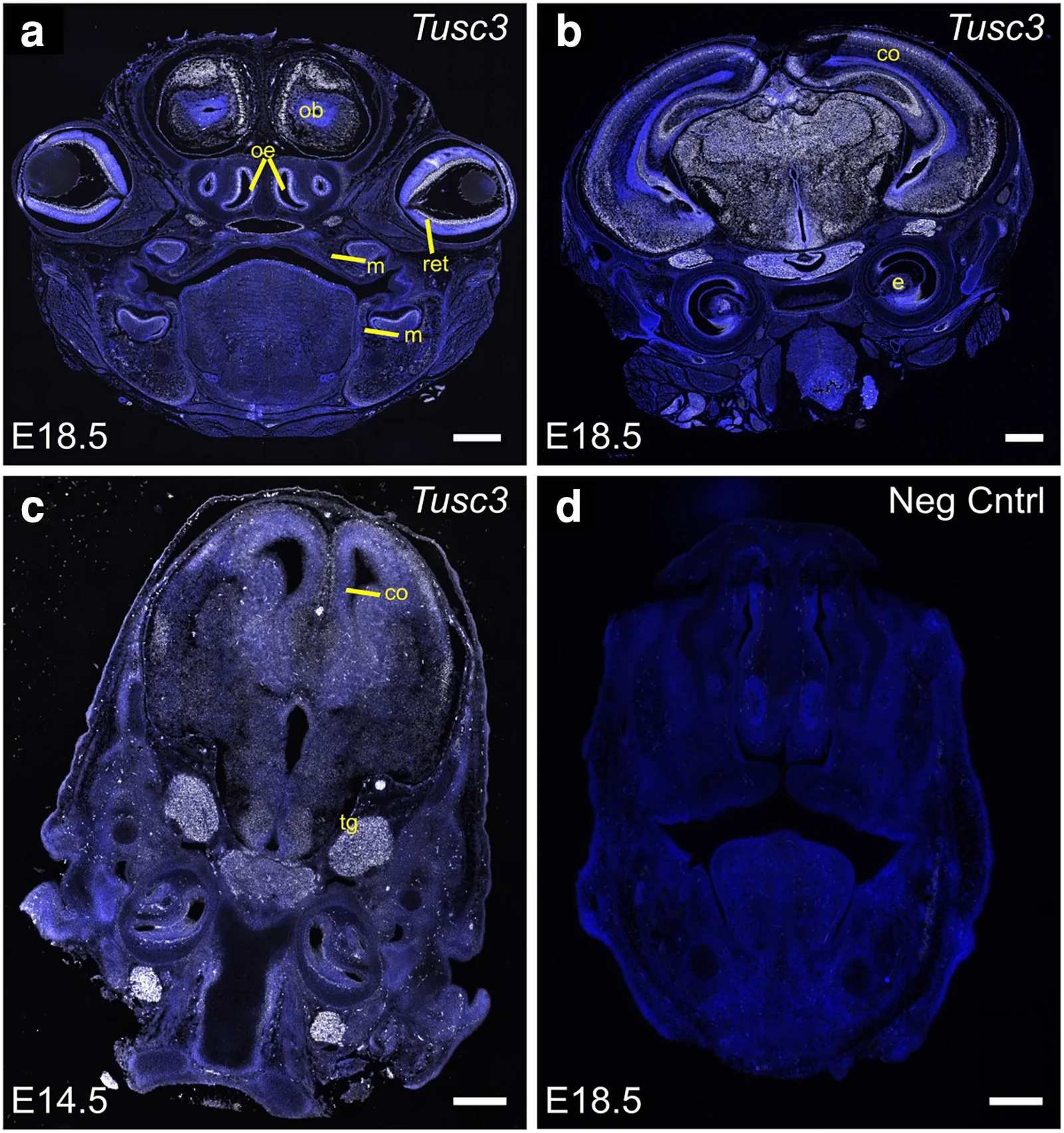
*Tusc3* expression during embryonic development. a to c) RNAscope showing the *Tusc3* RNA expression (pseudo-colored in white) overlayed on DAPI (blue) in wildtype embryos is widespread throughout the brain, retina, olfactory epithelia, molar tooth bud, trigeminal ganglia, and in the ear region at E18.5 (a, b) and E14.5 (c) and negative control (d) showing a lack of signal, indicating the specificity of the *Tusc3* probe. co, cortex; e, ear; ob, olfactory bulb; oe, m, molar tooth bud; olfactory epithelia; ret, retina; tg, trigeminal ganglia. Scale bar, 500 µm, *n* = a minimum of 3 at each stage.

### *Tusc3* mutants exhibit preweaning lethality but no difference in body weights

The clinical presentation of our *TUSC3* patient, *Tusc3* expression during development, and our analysis of the IMPC data prompted us to further explore the role of *Tusc3* in craniofacial development. We brought the *Tusc3^del^* mice from the MMRC (C57BL/6N-*Tusc3^em1(IMPC)J^*/Mmucd; Stock number: 050255-UCD) into our colony. Since the IMPC data indicated a preweaning lethality in the *Tusc3^del/del^* mutants, we intercrossed the *Tusc3^del/wt^* mice and monitored the survival of the offspring at E18.5, postnatal day (P)1, P8, and P14. At E18.5, *Tusc3^del/del^* embryos were present at the expected Mendelian frequency (*n* = 63/234; *P* = 0.707; Fig. 4a). While mutants were also represented at near-expected levels at P1 (*n* = 5/27; *P* = 0.404; Fig. 4a), the survival rate decreased by P8 (4/41; *P* = 0.063), and dropped more dramatically at P14 (*n* = 11/131; *P* = 2.1 × 10^−5^; Fig. 4a). Thus, our data are consistent with the IMPC data and indicate that *Tusc3* deficiency is associated with reduced postnatal survival.

**Fig. 4.**
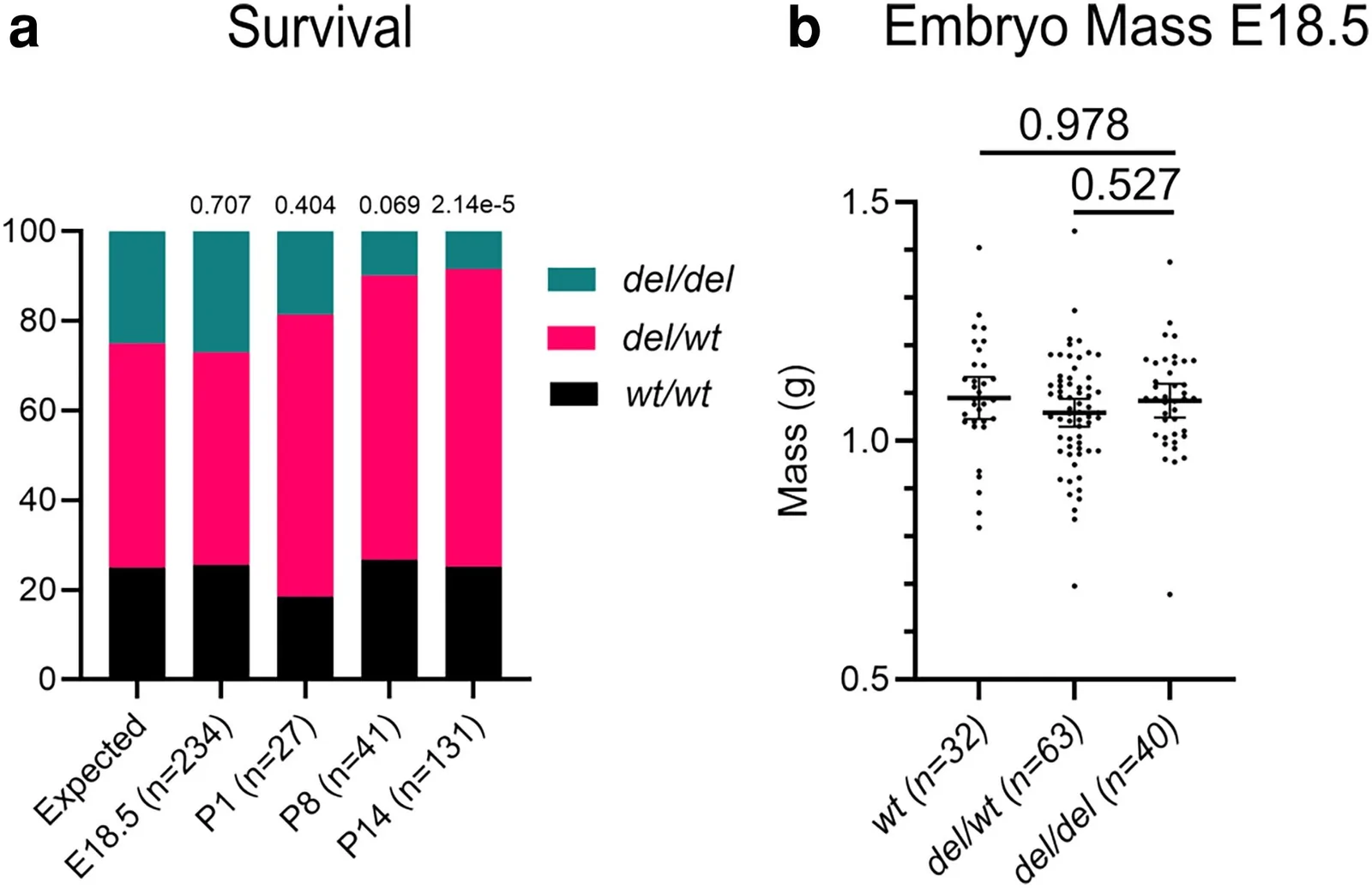
*Tusc3^del/del^* mutants exhibit preweaning lethality but do not show a significant change in body mass. Survival data (a) from the intercross of *Tusc3^del/wt^* mice at indicated stages. Body mass (b) is not altered in the *Tusc3^del/del^* embryos at E18.5 (*n* = 40).

Although the IMPC data suggested an abnormal body size at E18.5, our measurements of embryo weights at this stage revealed no significant difference between *Tusc3^del/del^* embryos and their littermate *Tusc3^wt/wt^* wildtype or *Tusc3^del/wt^* heterozygote controls (*n* = 40; *P* = 0.978; Fig. 4b). Taken together, these data demonstrate that while *Tusc3* is critical for postnatal survival, it has no significant effect on overall embryo mass at E18.5.

### *Tusc3* deletion phenotype and RNA-seq for differential gene expression

We intercrossed the *Tusc3^del/wt^* mice and observed the craniofacial phenotype of *Tusc3^del/del^* embryos at E18.5. Only one out of 21 *Tusc3^del/del^* embryos displayed a short mandible resulting in an open mouth phenotype, while the rest of the *Tusc3^del/del^* embryos looked comparable to the littermate controls (Fig. 5, a and b). We examined the palatal phenotype of *Tusc3^del/del^* embryos at E18.5 after removing the mandible. Out of the 21 *Tusc3^del/del^* embryos, one mutant embryo exhibited a high-arched palate (Fig. 5, c and d). However, the rest of the *Tusc3^del/del^* embryos exhibited normal palatal structures. To further characterize the mandible phenotype in the *Tusc3^del/del^* mutants, we carried out skeletal preparations to highlight cartilage and bone and measured the length of the mandible relative to their head length. A larger sample of *Tusc3^del/del^* mutants did not exhibit a marked difference in mandible size compared to their littermate controls (*n* = 13; *P* = 0.9867; Fig. 5g). We also measured the area of the cortex in the *Tusc3* mutant brains at E18.5 and P1. *Tusc3^del/del^* embryos did not exhibit any significant difference in the cortical surface area at E18.5 (*n* = 8; *P* = 0.239; Fig. 5h). However, the *Tusc3^del/del^* mutants exhibited a slight decrease in the cortical area at P1 (*n* = 4; *P* = 0.045; Fig. 5, e, f and i).

**Fig. 5.**
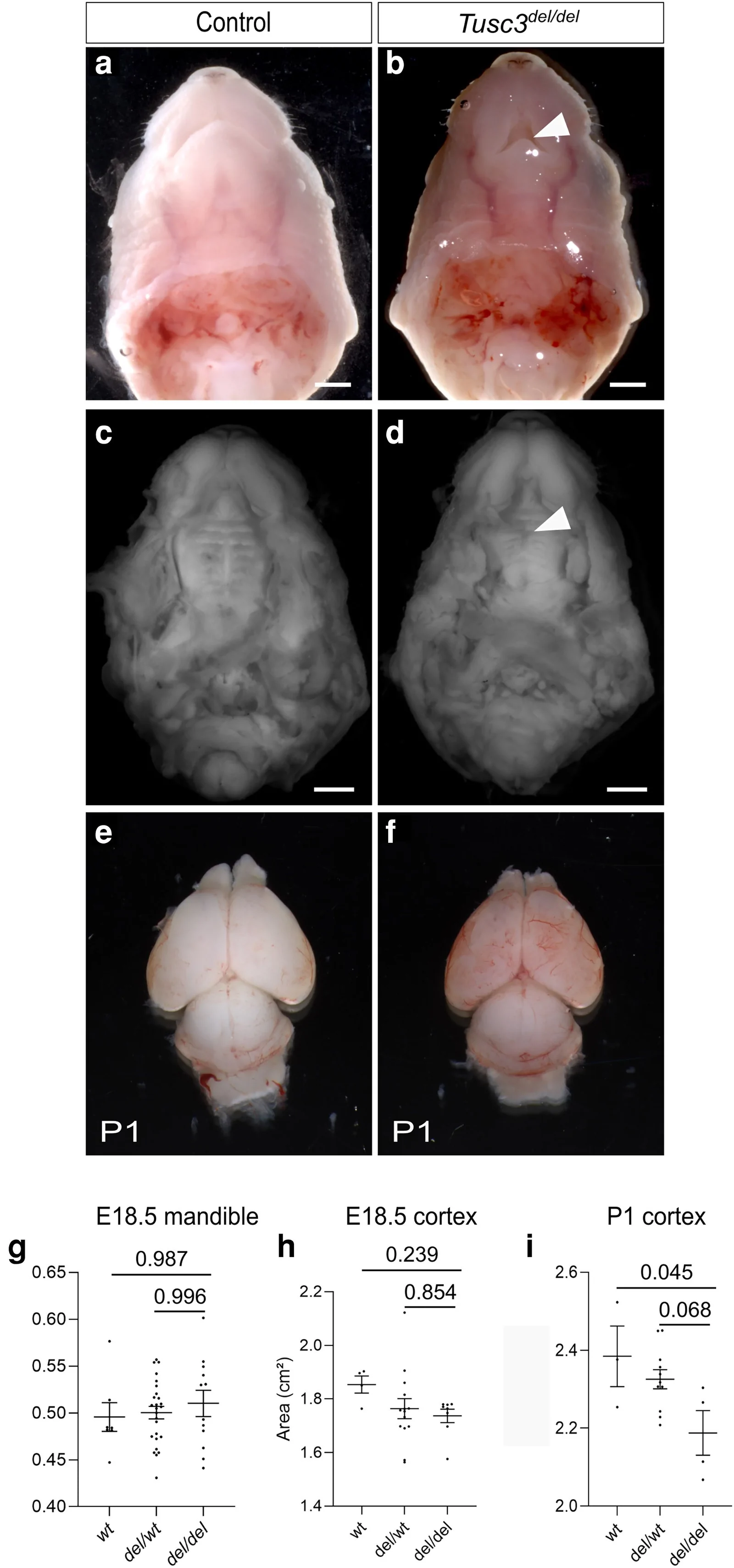
Mandible and cortical phenotype in *Tusc3^del/del^* mutants. a to d) Whole-mount ventral (a, b), and palatal (c, d) views of wildtype (a, c) and *Tusc3^del/del^* (b, d) embryos at E18.5. Arrowhead in b points to the short mandible resulting in an open mouth phenotype in *Tusc3^del/del^* mutants. Arrowhead in d points to the high arched palate in *Tusc3^del/del^* mutants. e, f) Whole-mount view of brains of wildtype (e) and *Tusc3^del/del^* (f) mutants at postnatal day 1 (P1). Measurement of mandible length proportional to the head length (g) from the skeletal preparations at E18.5 (*n* = 13). (h, i) Measurement of the cortex area from the whole-mount brain images at E18.5 (h) (*n* = 8) and P1 (i) (*n* = 4). Scale bar, 1 mm.

Finally, to identify any differentially expressed genes downstream of *Tusc3* that may help determine an underlying molecular mechanism, we performed bulk transcriptional profiling in facial and brain tissues of *Tusc3* mutants and wildtype littermate controls at E14.5 (*n* = 3). Other than *Tusc3*, we did not find any differentially expressed genes in the facial or brain tissues in *Tusc3^del/del^* mutants compared to wildtype (Supplementary Table 1). Volcano plots further demonstrated the limited transcriptional alterations observed in both brain and craniofacial tissues, with *Tusc3* representing the only significantly differentially expressed gene (Supplementary Fig. 1, a and b). Principal component analysis of normalized RNA-seq data demonstrated clear separation between brain and craniofacial samples, whereas mutant and control samples within each tissue exhibited substantial overlap, consistent with the limited transcriptional differences associated with TUSC3 deficiency (Supplementary Fig. 1c). To further assess whether coordinated transcriptional changes were present despite the limited number of differentially expressed genes, we performed rank-based pathway enrichment analysis using KEGG, Reactome, WikiPathways, and Gene Ontology Biological Process databases with all expressed genes from brain and craniofacial tissues. No significantly enriched pathways were identified in the brain dataset after correction for multiple testing (Supplementary Table 2). In the craniofacial dataset, no pathways were enriched at an adjusted *P*-value threshold of <0.05. However, 2 WikiPathways gene sets, Matrix Metalloproteinases and Endochondral Ossification, were enriched at the commonly used GSEA adjusted *P*-value threshold of <0.25 (Supplementary Table 3). Taken together, these data suggest that loss of *Tusc3* does not result in broad transcriptional dysregulation in embryonic brain or craniofacial tissues at E14.5. Although *Tusc3* is broadly expressed during embryonic development, craniofacial abnormalities associated with TUSC3 deficiency occurred at low penetrance in our mouse model.

Collectively, our analyses identify preweaning lethality as the most consistent phenotype associated with TUSC3 deficiency, while craniofacial abnormalities occur at low penetrance and cortical phenotypes remain modest.

## Discussion

With the increasing affordability of human genome sequencing, there has been a significant rise in the identification of variants of uncertain significance. In this study, we identified a patient with mutations in the *TUSC3* promoter region possibly associated with cleft palate. While *Tusc3^del/del^* mouse mutants exhibited mandible defects and cleft palate at a very low frequency, the majority of the mutants displayed normal morphology and mandible length. However, we did observe a slight reduction in the cortical area of these mutants. Although this cortical phenotype should be interpreted cautiously given the limited sample size at P1, it may suggest a subtle role for *Tusc3* during cortical development. The frequency of the phenotypes in the patients identified, the low penetrance in the mouse model, and the lack of transcriptional response suggest to us that *Tusc3* is unlikely to cause congenital anomalies on its own, but likely interacts with other pathogenic variants. Although RNA-seq analysis did not identify broad transcriptional dysregulation in embryonic brain or craniofacial tissues following loss of *Tusc3*, this does not exclude an important developmental role for TUSC3. TUSC3 functions as a component of the oligosaccharyltransferase (OST) complex and has established roles in protein N-glycosylation, ER homeostasis, magnesium regulation, and proteostasis. Therefore, the molecular consequences of TUSC3 deficiency may involve post-transcriptional or context-dependent mechanisms that are not readily captured by bulk transcriptomic analysis. The relatively subtle transcriptional and phenotypic changes observed in our study may therefore reflect incomplete penetrance and/or functional compensation rather than an absence of biological function. Together, our findings support a potential role for *Tusc3* in craniofacial and cortical development while suggesting that TUSC3 deficiency alone may be insufficient to consistently drive developmental abnormalities.

*Tusc3* was previously reported to be enriched in reproductive systems as well as in the adipose tissue, colon, cervix, and lung in human samples (Zhou and Clapham 2009). However, embryonic expression of *Tusc3* had not been rigorously interrogated. Here, we carried out RNA in situ hybridization using RNAscope probes and identified *Tusc3* expression in the developing brain, epithelial region in the head, neuronal tissues, and in developing tooth bud. This expression pattern is consistent with the role of *Tusc3* in various epithelial cancers (Vanhara et al. 2013; Kratochvilova et al. 2015; Feng et al. 2018). Furthermore, the localization of *Tusc3* in these specific embryonic tissues suggests that it may influence cellular pathways common to both development and oncogenesis.

We analyzed the *Tusc3^del/del^* mutant phenotype data from the IMPC and noted cleft palate and aglossia of the tongue with severe hypoplasia of the mandible. In our breeding scheme of the *Tusc3^del/del^* mutants, we only observed one mutant out of 21 that exhibited a short mandible. Thus, *Tusc3* may have a role as a contributing pathogenic locus in craniofacial development but is unlikely to be a highly penetrant monogenic disease locus. Given the facial dysmorphism and cleft lip/palate in patients with *TUSC3* variants together with mouse data from here and IMPC data, it is plausible that *TUSC3* could be a risk locus that predisposes toward craniofacial and cortical malformations.

Although *Tusc3^del/del^* mutants exhibited significant preweaning lethality, the relatively low penetrance of overt craniofacial abnormalities suggests that craniofacial defects alone are unlikely to fully account for the survival phenotype. *Tusc3* was broadly expressed in embryonic neural and craniofacial tissues, raising the possibility that subtle neurological, developmental, or physiological abnormalities contribute to reduced postnatal viability. In particular, altered cortical development, impaired feeding behavior, or broader defects related to protein N-glycosylation, ER homeostasis, and proteostasis may collectively influence postnatal survival. Given the modest cortical phenotype and minimal transcriptional alterations observed in our study, additional molecular and functional analyses will be necessary to determine the mechanisms underlying preweaning lethality in *Tusc3* mutant mice.

*Tusc3* functions could be redundant with its paralogous family members. MAGT1 and TUSC3 are accessory subunits of the mammalian endoplasmic reticulum OST complex and are orthologous to the yeast OST proteins Ost3 and Ost6, respectively (Mohorko et al. 2014; Mohanty et al. 2020). In mammals, MAGT1 and TUSC3 share conserved sequence homology, similar transmembrane topology, and a luminal thioredoxin-like domain, consistent with an ancestral gene duplication event (Kelleher et al. 2003). Structural studies have shown that mammalian OST complexes incorporate either MAGT1 or TUSC3 in a mutually exclusive manner, analogous to the Ost3/Ost6 system in yeast (Mohorko et al. 2014).

Like *TUSC3*, pathogenic variants in *MAGT1* cause X-linked intellectual disability and developmental delay with mild facial dysmorphism (Blommaert et al. 2019). Given their close ancestral relationship, *Magt1* could partially compensate for loss of *Tusc3*, potentially contributing to reduced craniofacial phenotype penetrance in mice. Analysis of spatial transcriptomic data from the Mouse Organogenesis Spatiotemporal Transcriptomic Atlas revealed enrichment of *Magt1* in craniofacial regions overlapping with *Tusc3*, whereas *Magt1* expression was limited in developing embryonic brain regions (Chen et al. 2022). However, we did not observe significant differential expression of *Magt1* in our E14.5 RNA-seq dataset, suggesting that compensatory upregulation of *Magt1* does not occur at the transcriptional level in *Tusc3* mutant tissue. Instead, any potential compensation may occur through overlapping biological functions or post-transcriptional mechanisms that are not readily captured by bulk transcriptomic analysis. Together, these findings suggest partial functional redundancy in craniofacial and/or cortical development.

A limitation in interpreting the clinical findings from our study is the presence of an additional maternally inherited duplication at chromosome 1p34.3-p34.2 in the proband. Although the homozygous TUSC3 promoter variant is consistent with several previously reported neurodevelopmental features associated with TUSC3 deficiency, the contribution of the 1p34 duplication to the overall phenotype cannot be ruled out. Previous studies have linked copy number variations involving the 1p34 region with developmental delay, intellectual disability, autism spectrum disorder, hypotonia, and craniofacial dysmorphism (Kumar et al. 2010; Tokita et al. 2015; Jacher and Innis 2018). However, the mother carrying the deletion was not reported to have any of the phenotypes discussed here. These reports suggest that dosage-sensitive genes within this interval may contribute to neurodevelopmental abnormalities and variable craniofacial phenotypes. Furthermore, the promoter variant was not explicitly tested in an experimental setting.

The presence of both the homozygous TUSC3 promoter variant and the 1p34 duplication raises the possibility that the proband phenotype may reflect oligogenic or multifactorial mechanisms rather than a purely monogenic disorder. This interpretation is additionally supported by our mouse studies, in which craniofacial abnormalities were observed at very low penetrance. Together, these findings suggest that *TUSC3* deficiency alone may be insufficient to robustly drive craniofacial malformations but could instead function as a susceptibility or modifying factor in the presence of additional genetic or developmental modifiers.

More broadly, our findings highlight the challenges of assigning causality in individuals carrying multiple likely causal variants, particularly for variably expressive neurodevelopmental and craniofacial phenotypes. Future studies incorporating functional characterization of the *TUSC3* promoter variant and larger patient cohorts will be important for clarifying the relative contributions of *TUSC3* deficiency and additional genomic alterations to disease presentation.

## Supplementary Material

jkag224_Supplementary_Data

## Data Availability

The data underlying this article are available in the article and in its online Supplementary material or from the authors. The genome sequencing data have been deposited in dbGAP as part of study phs002997.v1.p1, and the individuals reported here are subjects 25, 26, and 27. The RNA sequencing data have been deposited in the GEO repository as study GSE335382. Supplemental material available at G3 online.
