# Supplementary material for: Involvement of tumor suppressor candidate 3 in mouse survival, craniofacial, and cortical development
Source: G3 (Bethesda). 2026 Aug 21;16(10):jkag224. doi: 10.1093/g3journal/jkag224 (PMC13643766; doi:10.1093/g3journal/jkag224)
Supplement: Supplementary file 1 [file jkag224_supplementary_data.zip › Supplemental_Material_Legends_G3-2026-406956.docx]

**Figure S1. RNA-seq analysis in brain and craniofacial tissues of *Tusc3^del/del^* mutants.** (A, B) Volcano plots showing differential gene expression analysis of E14.5 brain (A) and craniofacial (B) tissues from *Tusc3^del/del^* mutants relative to wildtype controls. Differential expression analysis was performed using DESeq2 with thresholds of log2 fold change ≥ 0.5 and adjusted p-value < 0.05. Dashed vertical lines denote the log2 fold-change cutoff (±0.5), and the dashed horizontal line denotes the significance threshold (adjusted p-value = 0.05). *Tusc3* was the only significantly differentially expressed gene identified in either tissue dataset. (C) Principal component analysis (PCA) plot of normalized RNA-seq expression data from E14.5 brain and craniofacial tissues obtained from *Tusc3^del/del^* mutants and wildtype littermate controls (n = 3 per group). Principal component 1 (PC1) accounts for 95% of the variance and separates brain and craniofacial samples, whereas principal component 2 (PC2) accounts for 3% of the variance. Within each tissue group, mutant and control samples exhibit substantial overlap, consistent with the limited transcriptional differences observed in the differential expression analysis.

**Table S1.**

Differentially expressed genes identified from E14.5 brain and craniofacial RNA-seq datasets.

**Table S2.**

Rank-based pathway enrichment analysis of E14.5 brain RNA-seq data.

**Table S3.**

Rank-based pathway enrichment analysis of E14.5 craniofacial RNA-seq data.
